# Exploring the Impact of Bioactive Compounds Found in Extra Virgin Olive Oil on NRF2 Modulation in Alzheimer’s Disease

**DOI:** 10.3390/antiox14080952

**Published:** 2025-08-02

**Authors:** Marilena M. Bourdakou, Eleni M. Loizidou, George M. Spyrou

**Affiliations:** Bioinformatics Department, The Cyprus Institute of Neurology and Genetics, Nicosia 2371, Cyprus; marilenab@cing.ac.cy (M.M.B.); elenil@cing.ac.cy (E.M.L.)

**Keywords:** Alzheimer’s disease, extra virgin olive oil, NRF2, *NFE2L2*, interactome, regulome

## Abstract

Alzheimer’s disease (AD) is a progressive neurodegenerative disorder marked by amyloid-β (Aβ) plaques, neurofibrillary tangles, blood–brain barrier dysfunction, oxidative stress (OS), and neuroinflammation. Current treatments provide symptomatic relief, but do not halt the disease’s progression. OS plays a crucial role in AD pathogenesis by promoting Aβ accumulation. Nuclear factor erythroid 2-related factor 2 (NRF2) is a key regulator of the antioxidant response, influencing genes involved in OS mitigation, mitochondrial function, and inflammation. Dysregulation of NRF2 is implicated in AD, making it a promising therapeutic target. Emerging evidence suggests that adherence to a Mediterranean diet (MD), which is particularly rich in polyphenols from extra virgin olive oil (EVOO), is associated with improved cognitive function and a reduced risk of mild cognitive impairment. Polyphenols can activate NRF2, enhancing endogenous antioxidant defenses. This study employs a computational approach to explore the potential of bioactive compounds in EVOO to modulate NRF2-related pathways in AD. We analyzed transcriptomic data from AD and EVOO-treated samples to identify NRF2-associated genes, and used chemical structure-based analysis to compare EVOO’s bioactive compounds with known NRF2 activators. Enrichment analysis was performed to identify common biological functions between NRF2-, EVOO-, and AD-related pathways. Our findings highlight important factors and biological functions that provide new insight into the molecular mechanisms through which EVOO consumption might influence cellular pathways associated with AD via modulation of the NRF2 pathway. The presented approach provides a different perspective in the discovery of compounds that may contribute to neuroprotective mechanisms in the context of AD.

## 1. Introduction

Alzheimer’s disease (AD) is a progressive neurodegenerative condition characterized by various pathological features, including the accumulation of amyloid-β (Aβ) plaques, the formation of neurofibrillary tangles, dysfunction of the blood–brain barrier (BBB), increased oxidative stress, and neuroinflammation [1]. Current therapeutic options for AD include monoclonal antibody drugs, acetylcholinesterase inhibitors, and N-methyl-D-aspartate (NMDA) antagonists. While these treatments offer some improvement in patients’ quality of life, they do not halt or reverse AD progression [2]. Consequently, ongoing research aims to pinpoint novel targets and strategies for AD prevention and modification.

Oxidative stress (OS), characterized by an imbalance in the body’s antioxidant mechanisms, contributes significantly to the onset and progression of AD by exacerbating the accumulation of Aβ and neurofibrillary tangles (NFTs) [3]. This pathological condition arises from the excessive production of free radicals, including reactive oxygen species (ROS), due to disrupted antioxidant mechanisms. ROS, when present in high concentrations, can affect cellular components such as DNA, proteins, and lipids in both the nucleus and mitochondria, leading to a breakdown in their normal functions and exacerbating OS [4]. Numerous studies have underscored the significant role of oxidative stress in driving AD progression [5,6].

The evolution of antioxidant defense mechanisms serves as a protective response against oxidative stress, with the transcription factor Nuclear factor erythroid 2-related factor 2 (NRF2) acting as a primary regulator. NRF2 governs a broad spectrum of antioxidant enzymes involved in neutralizing oxidative stress, and its role has been extensively explored in various disease contexts [7]. NRF2, which is the product of the *NFE2L2* gene, is a very important transcription factor that regulates the expression of a large number of genes related to oxidative stress, mitochondrial biogenesis, mitophagy, and mitochondrial function [8]. Furthermore, NRF2 plays a critical role in modulating the inflammatory response and maintaining cellular redox homeostasis, thereby offering cytoprotection against various diseases, including those associated with neurodegeneration. Given its pivotal roles, NRF2 and its closely associated proteins emerge as promising therapeutic targets in the battle against AD [9]. By modulating the network around NRF2, researchers aim to intervene in the pathological processes underlying AD, potentially offering new avenues for therapeutic intervention and disease management [10].

Strong adherence to the Mediterranean diet (MD) has been linked to improved cognitive function and a lower risk of mild cognitive impairment [11]. Key components of this diet, such as extra virgin olive oil (EVOO), are believed to contribute to these benefits [12]. Natural compounds are usually known for their minimal acute and chronic toxicity, making them potentially suitable for therapeutic use. NRF2 interactome/regulome represents a group of significant molecular targets for the advantageous impacts of natural compounds [13]. Polyphenols contribute to the activation of endogenous antioxidant defenses, in part by modulating transcription factors such as NRF2. This regulatory effect underscores their potential in supporting cognitive health and protecting against cognitive decline [14].

In this study, we developed a computational approach centered on bioactive compounds from EVOO, aiming to investigate how their potential beneficial effects in AD may be related to the modulation of NRF2 (or its corresponding gene *NFE2L2*), a key regulator of antioxidant defenses against oxidative stress.

A key component in the presented strategy is the identification of common gene signatures that include NRF2-associated genes from both transcriptomic data on both AD and EVOO consumption. Additionally, we incorporated GWAS analysis to explore potential genetic associations with Alzheimer’s disease linked to these common genes. We then employed chemical structure-based analysis to compare EVOO’s bioactive compounds with NRF2 activators. Enrichment analysis was conducted in order to investigate common biological functions between EVOO consumption, AD, and NRF2 modulation. We anticipate that our findings will provide a foundational basis for future research on and clinical investigations into Alzheimer’s disease.

## 2. Methods

### 2.1. Methodology Overview

**Figure 1:**
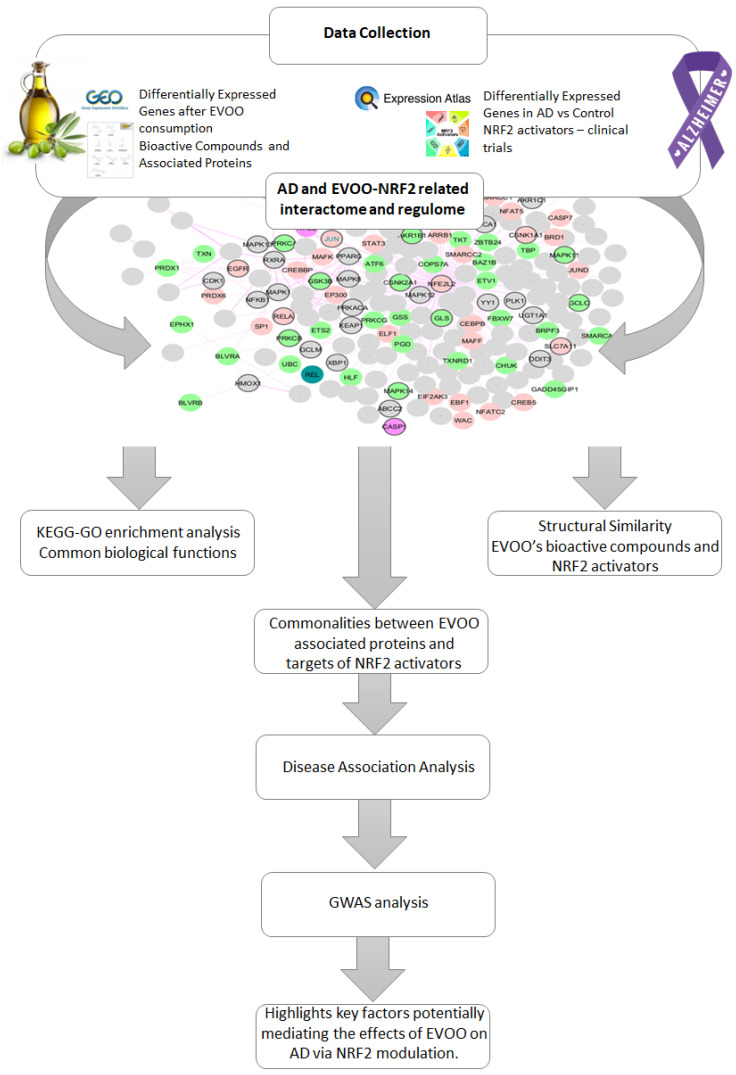
An overview of the multi-step computational workflow aimed at investigating the potential role of EVOO-derived bioactive compounds in modulating NRF2-related pathways in Alzheimer’s disease (AD).

The workflow of the proposed methodology is described in the following diagram (Figure 1). It describes data collection and a multi-step computational approach aimed at investigating the potential role of EVOO-derived bioactive compounds in modulating NRF2-related pathways in Alzheimer’s disease (AD). Gene expression data for NRF2 and its partners were retrieved from Expression Atlas (https://www.ebi.ac.uk/gxa/home, accessed on 1 January 2023) [15] to assess differential expression in AD, while EVOO-responsive gene profiles were obtained from GEO (https://www.ncbi.nlm.nih.gov/geo/, accessed on 3 March 2024) [16]. Bioactive compounds and their protein targets were collected from FooDisNET (http://140.113.120.248/FooDisNET/, accessed on 1 March 2024) [17] and CTD (http://ctdbase.org/, accessed on 1 March 2024) [18]. An NRF2-centered AD interactome was enriched with differentially expressed genes (DEGs) from AD and EVOO datasets. KEGG and GO enrichment analyses identified shared biological pathways among EVOO DEGs, AD-related NRF2 DEGs, and NRF2-targeting drugs. Structural similarity between EVOO compounds and known NRF2 activators was evaluated using pairwise Tanimoto scores. Overlapping targets between EVOO compounds and NRF2-activating drugs were analyzed for disease associations via the Enrichr web tool (https://maayanlab.cloud/Enrichr/, accessed on 20 September 2024) [19] and the OMIM database (https://omim.org/, accessed on 20 September 2024), and using the GWAS Catalog for AD-related genetic variants.

### 2.2. AD-Related NRF2 Interactome and Regulome

The AD-related NRF2 network was retrieved from our previous analysis [20]. More specifically, the NRF2 network was sourced from (http://sbi.imim.es/data/nrf2/, accessed on 1 January 2023) [21], where the authors compiled data on physical interactions among proteins involved in the NRF2 regulatory pathway from several sources. The total number of the NRF2-related genes involved in the network is 229. From these, the AD-related genes were found using information from the Expression Atlas—EMBL-EBI database (https://www.ebi.ac.uk/gxa/home, accessed on 1 January 2023) [15] (release 40), and precisely from the experiment with the accession number E-GEOD-5281—“Microarray analysis of six brain areas from Alzheimer’s disease patients and normal individuals” [22]—which was selected for its rich content of NRF2-related genes. Differentially expressed genes (DEGs) that consistently showed altered expression across all brain regions were selected for downstream analysis. Finally, 80 out of 229 were identified as DEGs based on the criteria of |log2FC| ≥ 1 with adj. *p*-values < 0.05.

### 2.3. EVOO’s Data Collection

Gene expression profiles of PBMCs in healthy subjects following the intake of a single 50 mL dose of high-polyphenol Italian extra virgin olive oil (EVOO) were obtained from the Gene Expression Omnibus database (GSE ID: GSE75025) [23]. The overall analysis was conducted using the R statistical environment (version: R-4.3.3) (http://www.R-project.org/, accessed on 3 March 2024). Utilizing the Limma R package (version 3.58.1) [24], a linear model was applied to generate moderated t-statistics from the gene expression data. Following dataset preprocessing, we conducted differential expression analysis, retaining DEGs with |log2FC| ≥ 0.5849 (fold change ≥1.5 or ≤0.67) and adj. *p*-values < 0.05. This threshold was selected because using a more stringent cutoff of |log2FC| ≥ 1 resulted in an insufficient number of genes for meaningful downstream analysis. Consequently, we identified 83 over-expressed and 98 under-expressed genes.

We also collected bioactive compounds of EVOO, with their structures in the form of the Simplified Molecular Input Line Entry Systems (SMILES) and their associated proteins, from Bourdakou et al., 2024 [25]. The whole list of bioactive compounds is presented in Table 1. More specifically, the bioactive compounds were retrieved from the literature and the corresponding structures from PubChem (https://pubchem.ncbi.nlm.nih.gov/, accessed on 20 March 2024) (version 1.8.1 beta) [26]. The available human proteins associated with the bioactive compounds of EVOO were obtained from the FooDisNET database [17], as well as from the Comparative Toxicogenomics Database (CTD) [18] (accessed in July 2024). Finally, we found 443 unique proteins associated with 6 EVOO bioactive compounds: Alpha-tocopherol, Apigenin, Caffeic acid, Hydroxytyrosol, Luteolin, and Pinoresinol.

SwissADME (http://www.swissadme.ch/, accessed on 8 June 2025) [27] was used to predict the physicochemical properties, pharmacokinetics, drug-likeness, and medicinal chemistry-friendliness of the bioactive compounds found in extra virgin olive oil (EVOO). Specifically, we obtained data on molecular weight, hydrogen bond donors and acceptors, lipophilicity (Log *p* values), solubility, topological polar surface area (TPSA), gastrointestinal absorption, blood–brain barrier permeability, and cytochrome P450 enzyme inhibition potential. These parameters helped us to assess the compounds’ potential bioavailability, permeability, and suitability as drug candidates. Comprehensive results from the SwissADME analysis for all the bioactive compounds are provided in Supplementary S1.

### 2.4. NRF2 Activator Collection

We gathered clinical trial drugs for AD with the capability to activate NRF2 from Osama et al., 2020 [28]. In particular, we compiled 13 drugs involved in various AD clinical trials that have been proven to activate NRF2 and 11 NRF2 activators, exhibiting beneficial effects on AD in in vivo models (Table 2). Furthermore, we collected 5 candidate repurposed drugs for AD which were found to activate a luciferase reporter for NRF2 activity in hippocampus-derived HT22 cells [20]. Finally, we collected 10 electrophilic activators of NRF2 that are in various stages of clinical development [29]. In total, we collected 34 unique NRF2 activators. The chemical structures of these NRF2 activators were retrieved from the Pubchem database, and the corresponding gene targets of each were retrieved from DrugBank (https://go.drugbank.com/, accessed on 1 April 2024) (version 5.1.12) [30] and from The Drug Repurposing Hub (https://repo-hub.broadinstitute.org/repurposing, accessed on 1 April 2024) (version: 3/24/2020) [31].

### 2.5. Structural Similarity

We converted the SMILES of the EVOO compounds, as well as of the NRF2 activators, into two single Structure Data File (SDF) library files using the OpenBabel software (version 2.4.1) [32]. Finally, by employing the Rcpi R package (version 1.38.0) [33], we computed the Tanimoto Similarity between the bioactive compounds present in EVOO and the NRF2 activators.

### 2.6. Genetic Investigation

We utilized the GWAS catalog database to identify potential genetic associations between Alzheimer’s disease and/or dementia and EVOO through NRF2, focusing on European populations only. A *p*-value < 5 × 10^−8^ was used to focus on associations of genome-wide significance between the genetic variants associated with the genes and the phenotypes of interest. We excluded variants where the effect size or the effect allele of the variant could not be identified either through the database or in the literature.

## 3. Results

### 3.1. NRF2-Related Genes Found in AD and EVOO

**Figure 2:**
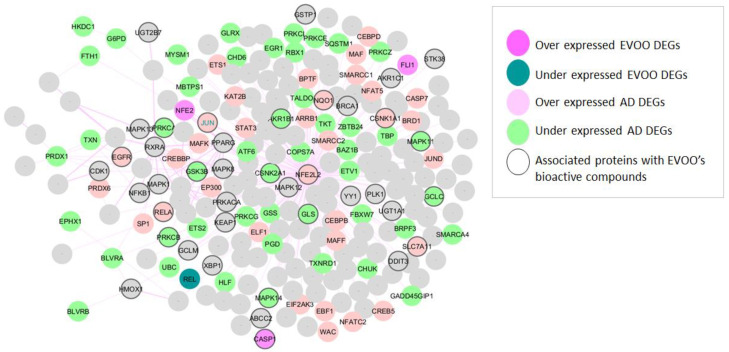
NRF2 interactome and regulome. Over-expressed AD DEGs are represented with pink color and AD under-expressed with green color. Over-expressed EVOO DEGs are represented with fuchsia color and under-expressed EVOO DEGs with petrol color. Proteins associated with the bioactive compounds of EVOO are presented with a black border.

The initial phase of this study involved identifying AD-related NRF2 and its partners that displayed differential expression following EVOO consumption. Figure 2 illustrates that among the 80 genes identified as differentially expressed in AD, 47 were under-expressed and 33 were over-expressed, including *NFE2L2*, which was found to be over-expressed. Furthermore, differential expression was observed in five NRF2 partners following EVOO consumption. Among these, three were found to be over-expressed (*FLJ1*, *NFE2*, *CASP1*), while two were under-expressed (*REL*, *JUN*). It is noteworthy that JUN is upregulated in AD and downregulated following EVOO consumption. DEGs in AD and EVOO are detailed in Supplementary S2.

Moreover, among the 443 human targets associated with six EVOO bioactive compounds (see Section 2.3 and Supplementary S3), 40 are also present in the NRF2 interactome and regulome (nodes with a black border in Figure 2). Among these, 16 (*NFE2L2*, *SLC7A11*, *GSK3B*, *NQO1*, *AKR1B1*, *GLS*, *EGFR*, *RELA*, *PRKCB*, *GCLC*, *CSNK1A1*, *MAPK11*, *MAPK14*, *CSNK2A1*, *PRKCA*, *and JUN*) exhibit differential expression in AD, including *JUN* and *NFE2L2*.

Importantly, *JUN* emerges as a key factor spanning all analytical pillars—being over-expressed in AD, downregulated following EVOO consumption, associated with EVOO’s bioactive compounds, and part of the *NRF2* interaction network.

### 3.2. Computational Insights into the Functional Landscape of the Protective Role of EVOO Consumption in AD

To delve deeper into the protective role of EVOO consumption in AD concerning NRF2, we compared the molecular mechanisms and biological processes (BPs) involving EVOO’s differentially expressed genes (DEGs) with NRF2-related AD DEGs and with the functional terms targeted by clinical trial drugs for AD with NRF2 activation ability or drugs used to treat other diseases. We utilized the web tool Enrichr, employing the KEGG 2021 HUMAN version and the GO Biological Process (GO-BPs) 2023 databases (https://maayanlab.cloud/Enrichr/, accessed on 2 May 2024) [19], with a selection criterion of an adj. *p*-value < 0.05, to identify significantly enriched pathways and terms.

Our analysis revealed 12 KEGG pathways and 47 GO-BP terms associated with EVOO, and 106 KEGG pathways and 404 GO-BP terms linked to NRF2 activators. Among these, eight KEGG pathways were shared between EVOO and NRF2 activators, including the C-type lectin receptor signaling pathway, the NOD-like receptor signaling pathway, Legionellosis, the Chemokine signaling pathway, Kaposi sarcoma-associated herpesvirus infection, Salmonella infection, the NF-κB signaling pathway, and Necroptosis.

Similarly, 26 GO-BP terms were found to be common between EVOO and NRF2 activators, such as Cellular Response To Lipopolysaccharide, Positive Regulation Of DNA-templated Transcription, Positive Regulation Of Cytokine Production, Cellular Response To Lipid, Regulation Of Transcription By RNA Polymerase II, Regulation Of Erythrocyte Differentiation, Cellular Response To Cytokine Stimulus, Positive Regulation Of Nucleic Acid-Templated Transcription, Regulation Of Interleukin-8 Production, Positive Regulation Of Transcription By RNA Polymerase II, Cellular Response To Peptide Hormone Stimulus, and Regulation Of Blood Vessel Endothelial Cell Migration.

**Figure 3:**
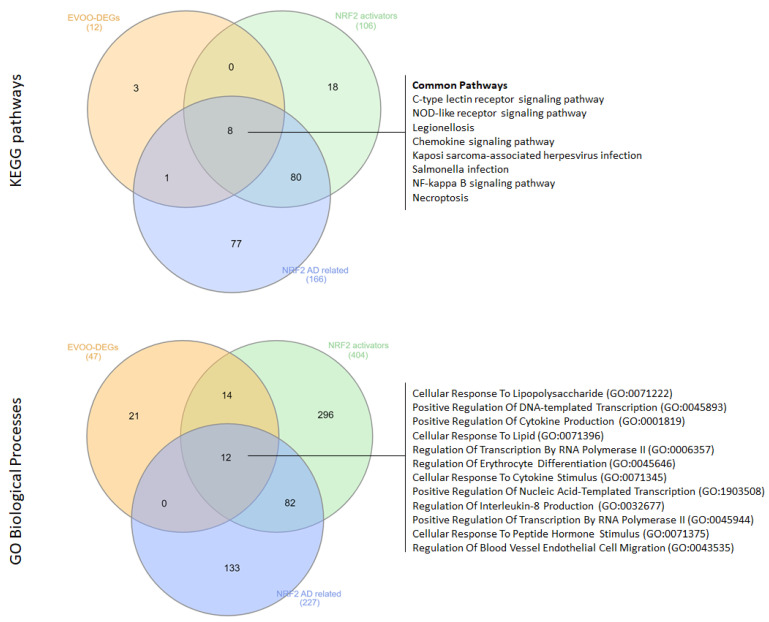
Venn diagrams illustrating the overlapping KEGG pathways and GO-BPs among EVOO DEGs, gene targets of NRF2 activators, and NRF2-related AD DEGs.

Furthermore, we investigated the common KEGG pathways and GO-BPs among EVOO, NRF2 activators, and functional terms significantly enriched from the analysis of NRF2-related AD DEGs. Notably, we found that the eight KEGG pathways were also significantly enriched in the analysis of AD DEGs. Additionally, 12 out of 26 GO-BP terms were shared among all conditions, including Cellular Response To Lipopolysaccharide, Positive Regulation Of DNA-templated Transcription, Positive Regulation Of Cytokine Production, Cellular Response To Lipid, Regulation Of Transcription By RNA Polymerase II, Regulation Of Erythrocyte Differentiation, Cellular Response To Cytokine Stimulus, Positive Regulation Of Nucleic Acid-Templated Transcription, Regulation Of Interleukin-8 Production, Positive Regulation Of Transcription By RNA Polymerase II, Cellular Response To Peptide Hormone Stimulus, and Regulation Of Blood Vessel Endothelial Cell Migration (Figure 3).

### 3.3. Structural Investigation of EVOO’s Bioactive Compounds and Drugs with NRF2 Activation Ability Under Ongoing Clinical Trial Investigation

**Figure 4:**
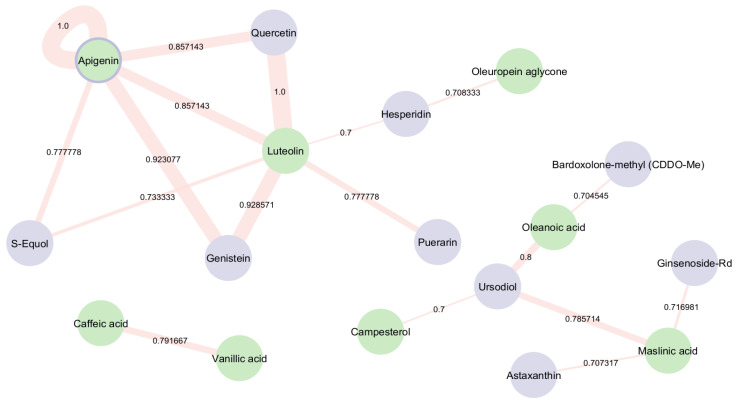
Similarity network representing the structural similarity between EVOO’s bioactive compounds and NRF2 activators in ongoing clinical trials. Green nodes illustrate the bioactive compounds and blue nodes illustrate the NRF2 activators, respectively. The edge width represents the Tanimoto similarity between compounds.

The bioactive compounds within EVOO were screened for potential structural similarity to known NRF2 activators using pairwise comparisons, with a Tanimoto coefficient threshold of 70%, which is a structural similarity score that indicates a sufficient number of compound connections. Figure 4 shows that Apigenin, a bioactive compound present in EVOO, is also listed among the clinical trial drugs that are known to activate NRF2 and have demonstrated positive effects on AD in in vivo models [28]. The self-loop around Apigenin highlights its dual role, indicating that it appears in both groups being compared—EVOO bioactive compounds and NRF2-activating trial drugs. Additionally, Luteolin, another bioactive compound in EVOO, shares structural identity with Quercetin, a plant flavonol from the flavonoid group of polyphenols, that is found in many fruits, vegetables, leaves, etc. Quercetin is currently involved in clinical trials for AD and has demonstrated NRF2 activation ability. Furthermore, Genistein—a naturally occurring isoflavone that is currently under investigation in clinical trials for AD—exhibits a high degree of structural similarity (Tanimoto score > 0.9) with the EVOO-derived compounds Apigenin and Luteolin. This strong structural alignment may suggest common pharmacophore features or shared biological activities, particularly in relation to NRF2 activation.

Ursodiol, an FDA-approved medication for primary biliary cirrhosis, likely enhances therapeutic efficacy by upregulating NRF2, thereby inducing detoxification and antioxidant mechanisms. It was found to be structurally similar to three other bioactive compounds: Oleanoic acid, Campesterol, and Maslinic acid. Oleanoic acid was also found to be similar to Bardoxolone-methyl (CDDO-Me). Additionally, Maslinic acid displayed structural similarity with Astaxanthin and Ginsenoside-Rd, while Luteolin exhibited similarity with Puerarin and Hesperidin. These aforementioned NRF2 activators are currently undergoing clinical trials for other diseases, showing positive effects on AD in in vivo models.

Moreover, Luteolin and Apigenin were found to share structural similarity with S-Equol, a compound participating in AD clinical trials that has been proven to activate NRF2. Lastly, Oleuropein aglycone was identified to be structurally similar to Hesperidin.

### 3.4. Identifying Commonalities Between Proteins Associated with EVOO Bioactive Compounds and Targets of NRF2-Activating Drugs

**Figure 5:**
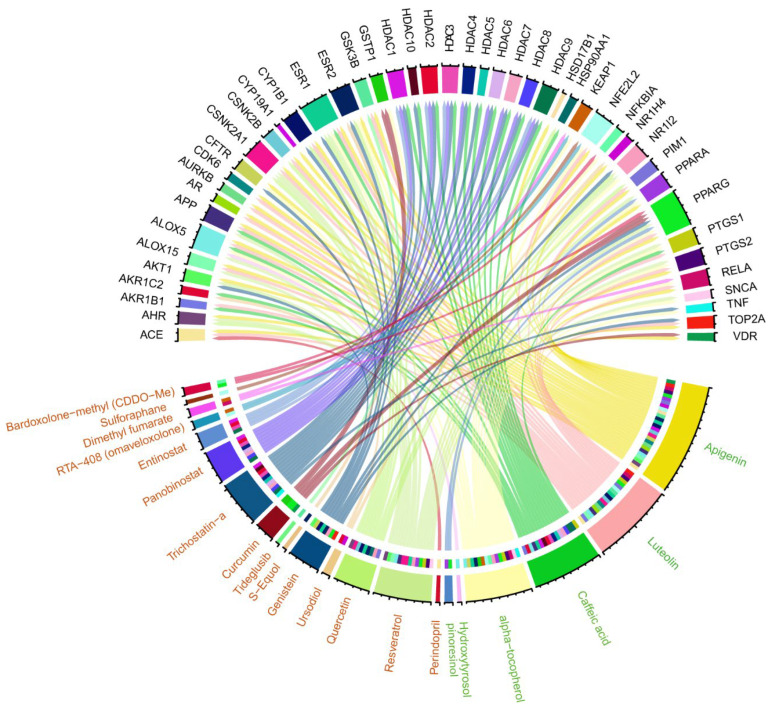
A Circos plot that summarizes the commonalities between proteins associated with EVOO’s bioactive compounds (green color) and targets of NRF2 activators in ongoing clinical trials (orange color).

To further explore the impact of EVOO on AD via NRF2 modulation, we compared the proteins associated with EVOO’s bioactive compounds to the gene targets of clinical trial drugs known to activate NRF2 (Table 2). This comparison revealed 47 common genes between the two groups (Figure 5, Supplementary S4). More specifically, six of EVOO’s bioactive compounds (Luteolin, Caffeic acid, alpha-tocopherol, Hydroxytyrosol, pinoresinol, and Apigenin) shared common associated proteins with the gene targets of 16 NRF2 activators, including Apigenin, namely Perindopril, Resveratrol, Quercetin, Ursodiol, Genistein, S-Equol, Tideglusib, Curcumin, Trichostatin-a, Panobinostat, Entinostat, RTA-408 (omaveloxolone), Dimethyl fumarate, Sulforaphane, Bardoxolone-methyl (CDDO-Me), and Apigenin. All commonalities are presented in Figure 5. We further investigated the biological functions in which the common genes are involved. The Enrichr web tool was used in order to find the statistically significant enriched KEGG pathways and GO-BPs (adj. *p*-values < 0.05). We ended up with 111 significant KEGG pathways and 589 GO-BPs. It is worth noting that the eight KEGG pathways that were found to be common between EVOO DEGs, NRF2 activators, and NRF2-related AD DEGs are also included in the 111 significant KEGG pathways. Following the same procedure, for the case of GO-BPs, 11 out of 12 are also included in the 589 significant GO-BPs: Regulation Of Transcription By RNA Polymerase II, Positive Regulation Of DNA-templated Transcription, Positive Regulation Of Transcription By RNA Polymerase II, Positive Regulation Of Nucleic Acid-Templated Transcription, Cellular Response To Lipid, Cellular Response To Peptide Hormone Stimulus, Regulation Of Blood Vessel Endothelial Cell Migration, Cellular Response To Cytokine Stimulus, Positive Regulation Of Cytokine Production, Cellular Response To Lipopolysaccharide, and Regulation Of Interleukin-8 Production.

We extended our investigation to explore the diseases associated with the 47 common genes. Using the Enrichr web tool and the OMIM database, we identified the most significantly enriched diseases linked to these genes. Table 3 illustrates that dementia emerges as the most significantly enriched disease, followed by migraine, myocardial infarction, asthma, and Alzheimer’s disease.

### 3.5. Genetic Associations of the Common Genes Between the Associated Proteins of EVOO and Targets of NRF2 Activators in Ongoing Clinical Trials with Alzheimer’s Disease

We utilized the GWAS catalog database to identify potential genetic associations with Alzheimer’s disease and/or dementia linked to the 47 common genes present between associated proteins of EVOO and targets of NRF2 activators in ongoing clinical trials. Our genetic investigation revealed a single-nucleotide polymorphism (SNP) (rs2154481-C) in the *APP* locus associated with protective effects on Alzheimer’s disease (OR = 0.95, *P* = 1 × 10^−11^). We sought to functionally explore the protective variant to uncover its potential association with gene expression in brain tissues, using Mayo and ROSMAP studies [34], which are currently the ones offering AD gene expression data. Specifically, we investigated the cerebellum (CER) region from the Mayo study and cortical regions from both studies. Based on our findings, the SNP was also found to be an expression quantitative trait locus (eQTL). The common allele that lowers the risk for AD is also linked to lower *APP* expression in the cerebellum, cerebral cortex, and temporal cortex of AD patients. This specific allele was also found to be enriched in cognitively healthy centenarians [35].

## 4. Discussion

The Mediterranean diet has been linked to enhanced cognitive function and a reduced risk of dementia. Key components of this diet, such as EVOO, contain polyphenols that contribute to these benefits [11]. Polyphenols exert their effects by activating endogenous antioxidant defense mechanisms, including the regulation of transcription factors like NRF2 [14]. NRF2 has garnered increasing attention due to its protective effects against various neurodegenerative diseases [9]. Several studies, both in vitro and in vivo, have demonstrated NRF2’s involvement in reducing oxidative stress in AD, thereby slowing disease progression. NRF2 activation has been shown to increase the expression of heme oxygenase-1 (HO-1) in mice treated with Aβ1-42, a key enzyme known for its anti-neuroinflammatory effects and its ability to protect neurons from cell death induced by neurotoxins. This effect has been linked to improvements in cognitive deficits in mice [36].

In this study, we developed a computational methodology to investigate the potential health benefits of EVOO’s bioactive compounds on AD through modulation of NRF2 interactome and regulome. In the first phase, we identified NRF2-associated genes that are differentially expressed in AD and following EVOO consumption. Notably, *JUN* was found to be significantly over-expressed in AD, with a log_2_ fold change (log_2_FC) of +1 and an adj. *p*-value < 0.05, based on Expression Atlas data. In contrast, following EVOO consumption, *JUN* was significantly downregulated, with a log_2_FC of −0.637 and an adj. *p*-value < 0.05, highlighting an opposite transcriptional response to dietary intervention. This regulation may be partially attributed to alpha-tocopherol—a key bioactive compound in EVOO—or to other EVOO-derived polyphenols such as hydroxytyrosol and oleuropein, which have been associated with the modulation of signaling pathways involving *JUN* expression [37]. Elevated c-*JUN* expression has been linked to neurodegeneration in AD, and its inhibition has been shown to reduce neuronal death in in vivo AD models [38]. While EVOO is a major and consistently consumed source of polyphenols in the Mediterranean diet, other components contribute additional compounds that can also modulate *JUN* expression—albeit being components that are typically consumed less frequently or in smaller quantities. For instance, resveratrol, found in red wine, has been shown to inhibit the JNK/c-JUN pathway and reduce neuroinflammation in AD models [39]. Similarly, anthocyanins from berries suppress *JUN*-related inflammatory signaling [40], and epigallocatechin gallate (EGCG), a catechin in green tea, downregulates both *JUN* expression and JNK activity in neuronal cells [41]. However, the consumption of red wine, berries, and green tea is generally less regular or abundant compared to the consumption of EVOO, which is a dietary staple used daily in Mediterranean cuisines. Therefore, the consistent intake of EVOO may offer a more sustained and cumulative effect on *JUN* modulation. The convergence of EVOO-derived and other polyphenol bioactives on shared inflammatory and stress response pathways supports their potential synergistic role in modulating AD-related symptoms.

Moreover, among the DEGs in AD, 16 are also associated with EVOO’s bioactive compounds. From these, 10 out of 16 are associated with Alpha-tocopherol. It has been reported that Alpha-tocopherol slows disease progression in patients with moderately severe impairment as a result of AD [42]. According to reports, Alpha-tocopherol treatment significantly reduces OS, restores NRF2 levels, and decreases inducible nitric oxide synthase (iNOS) levels, as demonstrated through immunocytochemistry [43].

In the second part of the study, we explored the protective effects of EVOO consumption on AD by examining the shared KEGG pathways and GO-BPs influenced by both EVOO and NRF2 activators from clinical trials that are also implicated in AD. We ended up with 8 common KEGG pathways and 12 GO-BPs. Among these, the NF-κB signaling pathway plays a significant role in AD as it is an important modulator of age-related disorders and aging [44]. Moreover, polyphenols modulate abnormal cellular signaling induced by pro-inflammatory stimuli and oxidative stress, such as that related to NF-κB signaling pathway [45]. Excessive activation of NF-κB has been specifically implicated in the neuropathological features of AD. Multiple studies have documented increased NF-κB activation in the brains of AD patients, particularly in the most affected brain regions [46]. In addition, our analysis revealed other pathways. These include the C-type lectin receptor signaling pathway and NOD-like receptor signaling pathway, both of which are crucial components of the innate immune system involved in pathogen recognition and the activation of inflammatory responses [47,48,49,50].

Complementing these pathway-level findings, the twelve common GO-BP terms enriched across EVOO, NRF2 activators, and AD-related DEGs highlight key cellular functions relevant to neuroprotection. Notably, terms such as “cellular response to lipopolysaccharide” and “cellular response to cytokine stimulus” underscore the involvement of immune and inflammatory signaling cascades [51,52]. Chronic inflammation is a hallmark of Alzheimer’s disease, contributing to neuronal dysfunction and accelerating disease progression. Cytokines—critical mediators of the immune response—play a central role in neuroinflammation and are increasingly recognized as key contributors to AD pathogenesis [53]. Moreover, the chemokine signaling pathway plays a key role in directing the migration of immune cells and modulating neuroinflammation, a recognized contributor to AD pathology [54,55]. Additionally, the enrichment of transcription-related terms, such as positive regulation of DNA-templated transcription and regulation of transcription by RNA polymerase II, suggests that EVOO and NRF2 activators may influence gene expression programs that are essential for cellular stress adaptation. Notably, transcription factors like TFII-I regulate RNA Polymerase II activity during both initiation and elongation phases, acting as key modulators of transcriptional responses to stress and contributing to cellular homeostasis [56].

In the third part of the study, we also examined the structural similarities between bioactive compounds in EVOO and drugs involved in clinical trials that are capable of activating NRF2 [20,28]. Notably, Apigenin, a compound found in EVOO, also functions as an NRF2 activator in clinical trials, showing positive effects on AD in animal models [28]. It has been reported that administering Apigenin to AD neurons alleviates neuronal hyper-excitability and reduces apoptosis. Additionally, experiments with activated inflammatory cells have been shown that Apigenin can suppress cytokine activation and nitric oxide (NO) production, thereby safeguarding AD neurons from inflammation-induced stress and preventing neurite retraction [57]. Additionally, Luteolin, another compound found in EVOO, has structural similarities to Quercetin, a flavonoid found in various foodstuffs, including olive oil, that has antioxidant properties, and which is currently involved clinical trials for AD due to its ability to activate NRF2 [28]. It has been reported that Quercetin reduces oxidative damage, regulates cytokine activity, prevents amyloid-beta clumping, and lowers tau protein phosphorylation [58,59,60]. It has also been reported that Luteolin has the potential to mitigate oxidative stress, neuroinflammation, apoptotic cell death and amyloidogenesis, and it can serve as a candidate for developing new therapeutic strategies to manage neurodegenerative conditions, particularly AD-like conditions [61]. Finally, an in silico study has highlighted ten phytochemicals from EVOO with the highest potential for activity against AD. These include Luteolin, Apigenin, and Squalene, offering valuable insights into the role of EVOO constituents in the treatment or prevention of AD [58].These compounds are indeed present in EVOO, though their concentrations vary depending on the olive variety, processing methods, and storage conditions [62]. Luteolin and apigenin are flavonoids that are found in modest amounts in EVOO, but they are also abundant in other Mediterranean diet staples such as celery and capsicum pepper [63]. While not exclusive to EVOO, their co-occurrence in the oil may contribute additive or synergistic effects in combination with other polyphenols such as hydroxytyrosol and oleuropein.

Furthermore, squalene, a triterpene hydrocarbon and key intermediate in cholesterol biosynthesis, is present in particularly high quantities in EVOO, with concentrations ranging from 200 to 7500 mg/kg depending on the olive cultivar, fruit ripeness, and processing conditions [64]. It constitutes over 90% of the hydrocarbon fraction in EVOO’s unsaponifiable matter, contributing significantly to its bioactive profile. While amaranth oil may contain comparable or even higher squalene levels (approximately 6000–8000 mg/kg) [65], it is not commonly consumed in Western or Mediterranean dietary patterns. Squalene is also found in other plant sources such as pumpkin seeds (89.0 mg/100 g) and quinoa (58.4 mg/100 g), though these levels are considerably lower than those in EVOO [66]. Shark liver oil contains the highest concentrations—up to 50–80% of the oil content [67]—but its use is limited due to ethical, environmental, and safety concerns. As such, EVOO remains the most widely consumed and nutritionally relevant source of squalene in the Mediterranean diet, offering a unique combination of lipid-soluble antioxidants with potential neuroprotective and anti-aging properties.

In the next phase of our study, we investigated the overlap between the proteins influenced by EVOO and the gene targets of ongoing clinical trials using NRF2 activators, revealing 47 common genes. Through enrichment analysis, we identified eight KEGG pathways shared among EVOO DEGs, NRF2 activators, and NRF2-related AD DEGs that were significantly enriched. Additionally, 11 out of 12 GO-BPs associated with these genes showed significant enrichment. Our disease enrichment analysis highlighted dementia and AD as the most significantly enriched conditions among this set of common genes. Finally, our genetic analysis highlighted rs2154481 in the *APP* locus as a protective variant against AD, reinforcing the potential role of *APP* regulation in disease susceptibility. Given that this SNP emerged from a targeted search among 47 common genes linked to EVOO-associated proteins and NRF2 activator targets, this suggests a mechanistic connection between *APP* expression, oxidative stress pathways, and neuroprotection. These findings support further investigation into how EVOO and NRF2 activation may modulate *APP* expression and Alzheimer’s pathology, particularly in individuals carrying this protective variant. APP was found to be associated with several EVOO-derived phenolic compounds, including Apigenin, Luteolin, and Caffeic acid. Apigenin has been shown to reduce BACE1 and β-CTF levels, leading to decreased Aβ deposition and improved cognition in APP/PS1 mice [68]. It also activates PI3K/Akt and Nrf2 signaling, while reducing neuroinflammation and oxidative stress [69]. It has also been reported that Luteolin reduced Aβ pathology in AD mice by modulating APP processing, inhibiting Aβ generation, and mitigating neuronal damage through a PPARγ-dependent mechanism [70]. Both Apigenin and Luteolin reduce neuroinflammation and oxidative stress by modulating NF-κB and activating Nrf2 signaling. Apigenin decreases Aβ production by downregulating BACE1 and β-CTF, while Luteolin inhibits Aβ generation and neuronal damage via PPARγ activation [71]. Caffeic acid has been reported to reduce APP and BACE1 expression, lowering Aβ1–42 levels in AD-like conditions [72]. Finally, Resveratrol—currently under investigation in AD clinical trials—not only activates NRF2, but also promotes non-amyloidogenic APP processing by enhancing α-secretase and suppressing β-secretase activity, further supporting its neuroprotective role [73].

Several neuroprotective mechanisms of phenolic compounds have been proposed, including antioxidant activity, reduction of tau aggregation, regulation of amyloidosis, modulation of neuroinflammation, and the ability to influence intracellular signaling pathways, particularly that of NRF2 [74,75]. NRF2 plays a pivotal role in cellular defense mechanisms against OS, and is critical for protecting against various neurodegenerative diseases, as it activates a range of protective genes through interaction with the antioxidant response element (ARE) [76,77,78]. Importantly, in some instances, OS alone may not sufficiently activate NRF2, necessitating external stimulation to enhance its protective functions [79]. Consequently, numerous studies have investigated various phenolic compounds to explore their potential as NRF2 activators.

Although our study is primarily computational, we aimed to enhance the translational relevance of our findings by evaluating the pharmacokinetic and drug-like properties of major EVOO-derived bioactive compounds. Using the SwissADME platform, we assessed key descriptors, including gastrointestinal (GI) absorption, blood–brain barrier (BBB) permeability, drug-likeness, and solubility. Among the compounds analyzed, tyrosol and pinoresinol demonstrated particularly favorable profiles, with high predicted GI absorption, BBB permeability, and acceptable bioavailability scores—features that support their potential as orally administered and CNS-targeting therapeutic agents (Supplementary S1).

However, translating such bioactive compounds from computational predictions to clinical use presents several challenges. First, effective dosing regimens remain unclear, as dietary compounds often require higher concentrations than typically obtained through nutrition to elicit therapeutic effects [80]. Additionally, variability in metabolic stability, compound degradation, and interactions with other dietary or pharmaceutical agents can influence efficacy [81]. These factors underscore the importance of in vitro and in vivo validation studies to determine optimal dosing, safety, and bioactivity under physiological conditions. Despite these limitations, our in silico analysis provides a valuable framework for prioritizing EVOO compounds in future experimental studies aimed at neuroprotection in AD.

While our study identifies important associations between certain EVOO compounds and neuroprotective effects through computational analyses and correlation-based methods, it is important to note that these findings do not establish direct causality. The predictions and associations reported are based on in silico methodologies, which can suggest potential mechanisms, but cannot definitively prove cause–effect relationships. Experimental validation in appropriate biological models is necessary to confirm the neuroprotective actions of these compounds. Therefore, our results should be interpreted as hypothesis-generating, providing a foundation for future mechanistic studies.

Another limitation of this study is the primary focus on the NRF2 pathway, which, although important in regulating oxidative stress responses, represents only one aspect of the complex and multifactorial nature of AD. AD involves various pathological processes, including mitochondrial dysfunction, abnormal protein aggregation (such as amyloid-beta plaques and tau tangles), microglial activation leading to neuroinflammation, synaptic loss, and other molecular alterations [82]. Therefore, while targeting the NRF2 pathway may offer therapeutic potential, it is crucial to consider that effective treatment of AD will likely require integrated approaches that address multiple pathological mechanisms simultaneously. This complexity should be acknowledged when interpreting our findings and planning future research directions.

In this study, we developed a computational framework to explore the potential molecular associations between bioactive compounds found in EVOO, the NRF2 signaling pathway, and AD. While our findings are based solely on in silico analyses and lack direct experimental validation, they are supported by the previous literature on the antioxidant and neuroprotective properties of specific EVOO bioactive compounds. By integrating transcriptomic data, compound–protein interactions, structural similarity, and enrichment analysis, we identified overlapping molecular features and biological processes that suggest possible mechanistic links among EVOO, NRF2 modulation, and AD-related pathways. These findings are intended to generate testable hypotheses and guide future experimental studies investigating the neuroprotective potential of EVOO-derived compounds acting through the NRF2 pathway.

## Figures and Tables

**Table 1:** Bioactive compounds of EVOO with their structures and their associated proteins.

Bioactive Compounds	Structures	Associated Proteins
Caffeic acid	C1=CC(=C(C=C1C=CC(=O)O)O)O	SULT1A3, SULT1C2, TP53, TYR, UBE2C, UGT1A1, UGT1A10, UGT1A3, UGT1A6, UGT1A7, UGT1A8, UGT1A9, UGT2B15, UGT2B17, UGT2B4, UGT2B7, MIF, KDM4E, MEN1, HDAC3, CA12, AKR1B10, KDM4A, SNUPN, ALDH1A1, EGFR, F2, CA1, CA2, LMNA, TTR, ESR1, MMP1, APP, GLA, POLB, CA3, HSP90AA1, HSP90AB1, MMP2, CYP3A4, ALOX5, GAA, MAPT, THRB, MMP9, AKR1B1, ARSA, HPGD, AKR1C4, PTPN1, NFKB1, ACHE, CA4, PTGS1, CA6, GPT, DPP4, APEX1, MPG, CA5A, PTPN7, AKR1C3, CA7, XDH, DUSP3, AKR1C2, HDAC4, ANTXR2, PMP22, KMT2A, AKR1C1, PTPN11, HYAL1, KCNH2, PLA2G7, HDAC1, KPNB1, CA9, HCAR2, HDAC7, HDAC2, HDAC10, HDAC11, EHMT2, HSD17B10, HDAC8, EGLN1, SLCO1B3, TDP1, HDAC6, POLK, BAZ2B, HDAC9, CA14, HDAC5, CA5B, SLCO1B1, P4HA1, PNLIP
Hydroxytyrosol	C1=CC(=C(C=C1CCO)O)O	ALOX5
Tyrosol	C1=CC(=CC=C1CCO)O	
Oleuropein aglycone	CC=C1C(C(=COC1O)C(=O)OC)CC(=O)OCCC2=CC(=C(C=C2)O)O	
Oleacein	CC=C(C=O)C(CC=O)CC(=O)OCCC1=CC(=C(C=C1)O)O	
Oleocanthal	CC=C(C=O)C(CC=O)CC(=O)OCCC1=CC=C(C=C1)O	
1-acetoxypinoresinol	CC(=O)OC12COC(C1COC2C3=CC(=C(C=C3)O)OC)C4=CC(=C(C=C4)O)OC	
Pinoresinol	COC1=C(C=CC(=C1)C2C3COC(C3CO2)C4=CC(=C(C=C4)O)OC)O	PPARG, PPARD, PPARA, ATAD5, RACGAP1
Apigenin	C1=CC(=CC=C1C2=CC(=O)C3=C(C=C(C=C3O2)O)O)O	TTR, DAPK1, CSNK2A1, TNKS2, KDM4E, SGK1, MEN1, PLK4, STK25, GABRP, CHEK1, GABRD, RGS12, NPC1, MAPK13, PDPK1, DAPK3, AKR1B10, ROCK2, GMNN, RPS6KA5, USP2, STK16, IDH1, PDE5A, USP1, STK10, GLS, CCNB2, TNKS, OXSR1, PAK4, CHEK2, ALDH1A1, EGFR, LMNA, ESR1, GBA, NR3C1, TP53, CYP1A1, CYBB, APP, MPO, CYP1A2, INSR, LCK, BCHE, CDK1, LYN, ABCB1, ELANE, CYP3A4, FGR, PARP1, ALOX5, ADORA3, CXCL8, AR, CYP2D6, MAPT, THRB, HSPA5, PIM1, TOP2A, VDR, CYP19A1, CYP2C9, CFTR, HSD17B1, PKM, CCNB1, GABRA1, ARSA, HPGD, ALOX15, TSHR, PRKCA, PRKACA, GABRG2, CSNK2A2, RXRA, NFKB1, MAOA, UGT1A1, UGT1A4, PRKACG, PRKACB, RPS6KB1, JAK1, CDK2, PON1, MAOB, MAPK3, DPP4, APEX1, GABRB3, MAPK1, AKT1, CYP2C19, ABCC1, PTGS2, OPRM1, UGT1A3, AHR, MAP2K2, FLT3, HSD17B2, PPARG, BRCA1, FEN1, THPO, OPRD1, OPRK1, CSK, SYK, MAPK8, MAPK9, GABRB2, XDH, CSNK1A1, CSNK1D, MAPKAPK2, CSNK1E, CLK1, CLK2, CLK3, RGS4, GSK3A, GSK3B, RORC, RPS6KA3, NEK2, MAP2K6, PLK1, MAPK12, MAPK10, PRKAA2, UGT2B15, BACE1, GNAI1, CSNK2B, HBB, GABRE, CSNK1G2, SMAD3, CDKL1, CDK6, CDK5, CDK16, NFKB2, RUNX1, PMP22, MAP2K1, TOP2B, KMT2A, PPARD, PTH1R, RELA, PPARA, DMPK, KCNMA1, STK4, PIN1, CAMK2B, CAMK2G, CAMK2D, DYRK1A, CBFB, CDK5R1, PDK1, STK38, RPS6KA1, MAPK11, NFE2L2, MAPK14, CAMK4, SMN1, MAPK6, HIF1A, CYP1B1, QPCT, NPSR1, PIM3, VRK2, CAMK1D, VRK3, MAPKAPK5, CCNB3, ESR2, ABCC2, PBK, CAMK1G, ATAD5, NR1H4, CAMKK2, MPHOSPH8, MAP3K5, ATXN2, HSD17B10, MYOC, VRK1, RIOK2, SLK, UGT1A10, UGT1A8, NEK6, CSNK1G1, SLCO1B3, NOX4, PAK6, TDP1, PIM2, PAK5, STK26, POLK, STK17A, TNIK, POLI, ABCG2, CAMK2A, POLH, NEU2, MAP4K5, SLCO1B1, CSNK1G3, HSD17B3, AURKB, DPP3, PTPN1, ACHE, CA1, CA2, CA3, CA4, ACE
Luteolin	C1=CC(=C(C=C1C2=CC(=O)C3=C(C=C(C=C3O2)O)O)O)O	TTR, PPARG, DAPK1, GALNT2, PBRM1, IPMK, TNKS2, KDM4E, MEN1, TNFRSF10B, ALOX15B, CA12, AKR1B10, KDM4A, GMNN, IDH1, USP1, GLS, CCNB2, SNUPN, TNKS, RAPGEF3, ALDH1A1, PLG, CA1, CA2, LMNA, ESR1, MMP1, GBA, NR3C1, TP53, CYP1A1, CYBB, APP, CYP1A2, LCK, BCHE, GLA, CDK1, POLB, ABCB1, ELANE, MMP2, MMP3, CYP3A4, PARP1, ALOX5, GAA, AR, BCL2, CYP2D6, MAPT, THRB, HSPA5, PIM1, TOP1, TOP2A, CYP2C9, CCNB1, TYR, MMP9, HPGD, ALOX15, TSHR, ALOX12, TFE3, CSNK2A2, NFKB1, MAOA, CA4, PTGS1, CDK2, PON1, MAOB, DPP4, APEX1, CD38, CYP2C19, PTGS2, FLT3, BRCA1, FEN1, MMP12, CA7, SYK, MMP13, RECQL, XDH, FASN, GSK3A, GSK3B, GALK1, BLM, RAN, CSNK2B, CSNK2A1, SMAD3, CDK6, CDK5, NFKB2, RUNX1, KMT2A, RELA, GLO1, BCL2L1, PIN1, CBFB, WRN, KPNB1, CDK5R1, PDK1, NFE2L2, SMN1, HIF1A, CYP1B1, CCNB3, EHMT2, ATAD5, ATXN2, HSD17B10, GPR35, NOX4, MBNL1, TDP1, POLK, BAZ2B, POLI, ABCG2, POLH, NEU2, AMY1A, MAPK14, MAPK10, PSIP1, AURKB, DPP3, CA3, ACE, AKR1B1, ACHE
Beta-sitosterol	CCC(CCC(C)C1CCC2C1(CCC3C2CC=C4C3(CCC(C4)O)C)C)C(C)C	
Campesterol	CC(C)C(C)CCC(C)C1CCC2C1(CCC3C2CC=C4C3(CCC(C4)O)C)C	
Squalene	CC(=CCCC(=CCCC(=CCCC=C(C)CCC=C(C)CCC=C(C)C)C)C)C	
Alpha-tocopherol	CC1=C(C2=C(CCC(O2)(C)CCCC(C)CCCC(C)CCCC(C)C)C(=C1O)C)C	CFTR, CLCN3, CPT1A, CRABP2, CRP, CXCL8, CYP2E1, CYP3A4, CYP3A5, CYP4F2, DDIT3, EIF2A, FN1, FYN, GCLC, GCLM, GPX1, GPX4, HBG2, HMOX1, HP, ICAM1, IKBKB, IL10, IL6, JUN, KCNH2, KEAP1, LCK, LDHA, LGALS3BP, LYN, MAPK1, MAPK14, MAPK3, MAPK8, MAPK9, MBP, MUC4, NFE2L2, NFKB1, NFKBIA, NOS2, NOS3, NQO1, NR1I2, PARK7, PARP1, PGK1, PLG, PPARA, PPARG, PTGS2, RELA, RPS6, RXRA, SELE, SLC1A2, SLC7A11, SLPI, SMAD4, SNCA, SOD1, TERT, TF, TNF, TNFRSF10B, TP53, VCAM1, XBP1, YY1, PRKCB, ALOX5, PRKCA, DGKA, PPP2CB, PPP2CA, SEC14L4, SEC14L3, SEC14L2, TTPA, AGTR2, EGFR, CYP1A2, DRD3, SLC6A3, CHRM1, OPRK1, ADRA2C, ADORA1, ADORA2A, ADRB3, ELANE, CCR4, HRH1, CTSG, PTGS1, CXCR2, TBXAS1, ADORA3, MMP9, PPP3CA, CYP2C9, CYP2D6, MC3R, NPY2R, MMP1, EDNRA, ESR2, UGT2B7, HTR6, TACR1, ESR1, HMGCR, CHRM4, OPRM1, CYP2C19, BDKRB2, SLC6A2, CALCR, AVPR1A, ADRA2B, DRD1, CASP1, CCKAR, CNR1, PTAFR, CHRM3, CA2, ADRA1D, ERBB2, LMNA, ADRB2, KMT2A, MEN1, CYP2A6, TACR2, VIPR1, SLC6A4, NR3C1, CCR2, PTPRC, HTR2A, USP1, HTR2B, HRH2, OPRD1, ADRB1, RAPGEF3, MC5R, CHRM5, DRD2, DRD4, NPY1R, MAPT, CCR5, ADRA2A, CHRM2, CYSLTR1, HTR2C, SIGMAR1, PDE5A, SNUPN, KPNB1, RAN, CXCR1, FLT1, MAOA, MC4R, PTPN1, GSTP1, NOX4
Oleanolic acid	CC1(CCC2(CCC3(C(=CCC4C3(CCC5C4(CCC(C5(C)C)O)C)C)C2C1)C)C(=O)O)C	
Maslinic acid	CC1(CCC2(CCC3(C(=CCC4C3(CCC5C4(CC(C(C5(C)C)O)O)C)C)C2C1)C)C(=O)O)C	

**Table 2:** Clinical trials and repurposed drugs with the capability to activate NRF2. Duplicate compounds are presented with the same colors.

Source	Compound Name	Smiles	Gene Targets
Clinical trials investigating drugs with NRF2 activation ability used in other diseases and demonstrating their positive effects on AD in vivo models	Dimethyl fumarate	COC(=O)C=CC(=O)OC	
	Artemether	CC1CCC2C(C(OC3C24C1CCC(O3)(OO4)C)OC)C	ATP1A1
	Ginsenoside-Rd	CC(=CCCC(C)(C1CCC2(C1C(CC3C2(CC(C4C3(CCC(C4(C)C)O)C)OC5C(C(C(C(O5)CO)O)O)OC6C(C(C(C(O6)CO)O)O)O)C)O)C)OC7C(C(C(C(O7)CO)O)O)O)C	
	Astaxanthin	CC1=C(C(CC(C1=O)O)(C)C)C=CC(=CC=CC(=CC=CC=C(C)C=CC=C(C)C=CC2=C(C(=O)C(CC2(C)C)O)C)C)C	NFKBIA
	Apigenin	C1=CC(=CC=C1C2=CC(=O)C3=C(C=C(C=C3O2)O)O)O	AKR1B1, AR, CDK6, CFTR, CYP19A1, CYP1B1, HSD17B1
	Hesperidin	CC1C(C(C(C(O1)OCC2C(C(C(C(O2)OC3=CC(=C4C(=O)CC(OC4=C3)C5=CC(=C(C=C5)OC)O)O)O)O)O)O)O)O	AURKB, CACNA1B
	Ebselen	C1=CC=C(C=C1)N2C(=O)C3=CC=CC=C3[Se]2	EPHX2, ALB
	Puerarin	C1=CC(=CC=C1C2=COC3=C(C2=O)C=CC(=C3C4C(C(C(C(O4)CO)O)O)O)O)O	
	Antroquinonol	CC1C(C(C(=C(C1=O)OC)OC)O)CC=C(C)CCC=C(C)CCC=C(C)C	
	Vanillic acid	COC1=C(C=CC(=C1)C(=O)O)O	
	Allicin	C=CCSS(=O)CC=C	TRPA1, TRPV1
Drugs with NRF2 activation ability demonstrated in AD clinical trials	DL-3-n-butylphthalide	O=C1C2=CC=CC=C2C(CCCC)O1	
	Resveratrol	C1=CC(=CC=C1C=CC2=CC(=CC(=C2)O)O)O	NQO2, CSNK2A1, PTGS1, PTGS2, ALOX15, ALOX5, AHR, PI4K2B, ITGA5, ITGB3, APP, SNCA, SIRT1, ESR1, MTNR1A, MTNR1B, CLEC14A, NR1I2, NR1I3, SLC2A1, CBR1, PPARA, PPARG, AKT1, KHSRP, YARS, CSNK2A1
	Curcumin	COC1=C(C=CC(=C1)C=CC(=O)CC(=O)C=CC2=CC(=C(C=C2)O)OC)O	PPARG, VDR, ABCC5, CBR1, GSTP1
	Sulforaphane	CS(=O)CCCCN=C=S	NFE2L2
	Lipoic Acid	C1CSSC1CCCCC(=O)O	LIPT1, SLC5A6, LIAS
	Perindopril	CCCC(C(=O)OCC)NC(C)C(=O)N1C2CCCCC2CC1C(=O)O	ACE, SFRP4
	S-Equol	C1C(COC2=C1C=CC(=C2)O)C3=CC=C(C=C3)O	ESR2, SHBG
	Doxycycline	CC1C2C(C3C(C(=O)C(=C(C3(C(=O)C2=C(C4=C1C=CC=C4O)O)O)O)C(=O)N)N(C)C)O	MMP8
	Quercetin	C1=CC(=C(C=C1C2=C(C(=O)C3=C(C=C(C=C3O2)O)O)O)O)O	PIK3CG, UGT3A1, ATP5A1, ATP5B, ATP5C1, PIM1, HIBCH, STK17B, ESR1, ESR2, NQO2, AHR, CYP1B1, ACTB, CSNK2A1, CSNK2B, EIF3F, HSP90AA1, HSPA2, RUVBL2, SF3B3, UBA1, SHBG, CBR1, CEBPB, NR1I2, HCK
	Genistein	C1=CC(=CC=C1C2=COC3=CC(=CC(=C3C2=O)O)O)O	NCOA1, ESR1, NCOA2, ESRRB, ESRRA, NR1I2, AKT1, GPER1, CYP1B1, SHBG, ESR2, TOP2A, PTK2B, CFTR, ESRRG, PPARG, TRPC5
	Tideglusib	C1=CC=C(C=C1)CN2C(=O)N(SC2=O)C3=CC=CC4=CC=CC=C43	GSK3B
	Pyridoxine	CC1=NC=C(C(=C1O)CO)CO	PDXK
	Benfotiamine	CC1=NC=C(C(=N1)N)CN(C=O)C(=C(CCOP(=O)(O)O)SC(=O)C2=CC=CC=C2)C	AGER
Selected electrophilic activators of NRF2 under clinical development	Bardoxolone-methyl (CDDO-Me)	CC1(CCC2(CCC3(C(C2C1)C(=O)C=C4C3(CCC5C4(C=C(C(=O)C5(C)C)C#N)C)C)C)C(=O)OC)C	NFKBIA, PPARG, STAT3
	RTA-408 (omaveloxolone)	CC1(CCC2(CCC3(C(C2C1)C(=O)C=C4C3(CCC5C4(C=C(C(=O)C5(C)C)C#N)C)C)C)NC(=O)C(C)(F)F)C	KEAP1, NFE2L2
	Dimethyl fumarate	COC(=O)C=CC(=O)OC	KEAP1, RELA
	ALKS-8700	COC(=O)C=CC(=O)OCCN1C(=O)CCC1=O	CHRNA10
	Oltipraz	CC1=C(SSC1=S)C2=NC=CN=C2	ANG
	Ursodiol	CC(CCC(=O)O)C1CCC2C1(CCC3C2C(CC4C3(CCC(C4)O)C)O)C	AKR1C2, NR1H4
	Sulforaphane	CS(=O)CCCCN=C=S	NFE2L2
	Curcumin	COC1=C(C=CC(=C1)C=CC(=O)CC(=O)C=CC2=CC(=C(C=C2)O)OC)O	PPARG, VDR, ABCC5, CBR1, GSTP1
	Resveratrol	C1=CC(=CC=C1C=CC2=CC(=CC(=C2)O)O)O	NQO2, CSNK2A1, PTGS1, PTGS2, ALOX15, ALOX5, AHR, PI4K2B, ITGA5, ITGB3, APP, SNCA, SIRT1, ESR1, MTNR1A, MTNR1B, CLEC14A, NR1I2, NR1I3, SLC2A1, CBR1, PPARA, PPARG, AKT1, KHSRP, YARS, CSNK2A1
	CXA-10	CCCCCCCCC(=CCCCCCCCC(=O)O)[N+](=O)[O−]	
Candidate repurposed drugs for AD with NRF2 activation ability	Curcumin	COC1=C(C=CC(=C1)C=CC(=O)CC(=O)C=CC2=CC(=C(C=C2)O)OC)O	PPARG, VDR, ABCC5, CBR1, GSTP1
	Trichostatin-a	CC(C=C(C)C=CC(=O)NO)C(=O)C1=CC=C(C=C1)N(C)C	HDAC1, HDAC10, HDAC2, HDAC3, HDAC4, HDAC5, HDAC6, HDAC7, HDAC8, HDAC9, CASP8, TNF
	Panobinostat	CC1=C(C2=CC=CC=C2N1)CCNCC3=CC=C(C=C3)C=CC(=O)NO	HDAC1, HDAC2, HDAC3, HDAC4, HDAC6, HDAC7, HDAC8, HDAC9
	Entinostat	C1=CC=C(C(=C1)N)NC(=O)C2=CC=C(C=C2)CNC(=O)OCC3=CN=CC=C3	HDAC1, HDAC2, HDAC3, HDAC9
	Parthenolide	CC1=CCCC2(C(O2)C3C(CC1)C(=C)C(=O)O3)C	

**Table 3:** Significantly enriched diseases of the 47 common genes between associated proteins of EVOO and targets of NRF2 activators in ongoing clinical trials.

*Disease*	*p*-Value	*q-Value*	*Genes*
*dementia*	2.64 × 10^−6^	5.27 × 10^−5^	[APP, TNF, SNCA]
*migraine*	0.000635	0.004234	[TNF, ESR1]
*myocardial infarction*	0.000635	0.004234	[ACE, ESR1]
*asthma*	0.001325	0.00655	[ALOX5, TNF]
*Alzheimer’s disease*	0.001695	0.00655	[APP, ACE]
*breast cancer*	0.001965	0.00655	[AKT1, ESR1]
*obesity*	0.002407	0.006876	[AKR1C2, PPARG]
*diabetes*	0.013451	0.033626	[ACE, PPARG]
*osteoporosis*	0.025555	0.049865	[VDR]
*ectodermal dysplasia*	0.025555	0.049865	[NFKBIA]
*fibrosis*	0.027846	0.049865	[APP, TNF, SNCA]
*ovarian cancer*	0.030132	0.049865	[TNF, ESR1]
*malaria*	0.032412	0.049865	[ACE, ESR1]

## Data Availability

The original contributions presented in the study are included in the article/Supplementary Materials.

## Supplementary Materials

The following supporting information can be downloaded at https://www.mdpi.com/article/10.3390/antiox14080952/s1: Supplementary S1: Pharmacokinetic and drug-likeness properties of EVOO bioactive compounds predicted by SwissADME; Supplementary S2: Differentially expressed genes in AD and EVOO; Supplementary S3: Proteins associated with EVOO’s bioactive compounds; Supplementary S4: Common genes between the proteins associated with EVOO’s bioactive compounds and the gene targets of clinical trial drugs with NRF2 activation ability.

## References

[1] Rajmohan R., Reddy P.H. (2017). Amyloid-Beta and Phosphorylated Tau Accumulations Cause Abnormalities at Synapses of Alzheimer’s disease Neurons. J. Alzheimer’s Dis..

[2] Breijyeh Z., Karaman R. (2020). Comprehensive Review on Alzheimer’s Disease: Causes and Treatment. Molecules.

[3] Zhao Y., Zhao B. (2013). Oxidative stress and the pathogenesis of Alzheimer’s disease. Oxid. Med. Cell. Longev..

[4] Guo C., Sun L., Chen X., Zhang D. (2013). Oxidative stress, mitochondrial damage and neurodegenerative diseases. Neural Regen Res..

[5] Bhatt S., Puli L., Patil C.R. (2021). Role of reactive oxygen species in the progression of Alzheimer’s disease. Drug Discov. Today.

[6] Smith M.A., Rottkamp C.A., Nunomura A., Raina A.K., Perry G. (2000). Oxidative stress in Alzheimer’s disease. Biochim. Biophys. Acta.

[7] Ngo V., Duennwald M.L. (2022). Nrf2 and Oxidative Stress: A General Overview of Mechanisms and Implications in Human Disease. Antioxidants.

[8] Kim J., Cha Y.N., Surh Y.J. (2010). A protective role of nuclear factor-erythroid 2-related factor-2 (Nrf2) in inflammatory disorders. Mutat. Res..

[9] Suzen S., Tucci P., Profumo E., Buttari B., Saso L. (2022). A Pivotal Role of Nrf2 in Neurodegenerative Disorders: A New Way for Therapeutic Strategies. Pharmaceuticals.

[10] De Plano L.M., Calabrese G., Rizzo M.G., Oddo S., Caccamo A. (2023). The Role of the Transcription Factor Nrf2 in Alzheimer’s Disease: Therapeutic Opportunities. Biomolecules.

[11] Petersson S.D., Philippou E. (2016). Mediterranean Diet, Cognitive Function, and Dementia: A Systematic Review of the Evidence. Adv. Nutr..

[12] Alkhalifa A.E., Al-Ghraiybah N.F., Kaddoumi A. (2024). Extra-Virgin Olive Oil in Alzheimer’s Disease: A Comprehensive Review of Cellular, Animal, and Clinical Studies. Int. J. Mol. Sci..

[13] Sidiropoulou G.A., Metaxas A., Kourti M. (2023). Natural antioxidants that act against Alzheimer’s disease through modulation of the NRF2 pathway: A focus on their molecular mechanisms of action. Front. Endocrinol..

[14] Martinez-Huelamo M., Rodriguez-Morato J., Boronat A., de la Torre R. (2017). Modulation of Nrf2 by Olive Oil and Wine Polyphenols and Neuroprotection. Antioxidants.

[15] Petryszak R., Keays M., Tang Y.A., Fonseca N.A., Barrera E., Burdett T., Fullgrabe A., Fuentes A.M., Jupp S., Koskinen S. (2016). Expression Atlas update--an integrated database of gene and protein expression in humans, animals and plants. Nucleic Acids Res..

[16] Edgar R., Domrachev M., Lash A.E. (2002). Gene Expression Omnibus: NCBI gene expression and hybridization array data repository. Nucleic Acids Res..

[17] Lin C.-Y., Lee J.-Y., Huang S.-H., Hsu Y.-C., Hsu N.-Y., Yang J.-M. FooDisNET: A database of food-compound-protein-disease associations. Proceedings of the IEEE 20th International Conference on Bioinformatics and Bioengineering (BIBE).

[18] Davis A.P., Wiegers T.C., Johnson R.J., Sciaky D., Wiegers J., Mattingly C.J. (2023). Comparative Toxicogenomics Database (CTD): Update 2023. Nucleic Acids Res..

[19] Xie Z., Bailey A., Kuleshov M.V., Clarke D.J.B., Evangelista J.E., Jenkins S.L., Lachmann A., Wojciechowicz M.L., Kropiwnicki E., Jagodnik K.M. (2021). Gene Set Knowledge Discovery with Enrichr. Curr. Protoc..

[20] Bourdakou M.M., Fernandez-Gines R., Cuadrado A., Spyrou G.M. (2023). Drug repurposing on Alzheimer’s disease through modulation of NRF2 neighborhood. Redox Biol..

[21] Cuadrado A., Manda G., Hassan A., Alcaraz M.J., Barbas C., Daiber A., Ghezzi P., Leon R., Lopez M.G., Oliva B. (2018). Transcription Factor NRF2 as a Therapeutic Target for Chronic Diseases: A Systems Medicine Approach. Pharmacol. Rev.

[22] Liang W.S., Dunckley T., Beach T.G., Grover A., Mastroeni D., Walker D.G., Caselli R.J., Kukull W.A., McKeel D., Morris J.C. (2007). Gene expression profiles in anatomically and functionally distinct regions of the normal aged human brain. Physiol. Genom..

[23] D’Amore S., Vacca M., Cariello M., Graziano G., D’Orazio A., Salvia R., Sasso R.C., Sabba C., Palasciano G., Moschetta A. (2016). Genes and miRNA expression signatures in peripheral blood mononuclear cells in healthy subjects and patients with metabolic syndrome after acute intake of extra virgin olive oil. Biochim. Biophys. Acta.

[24] Ritchie M.E., Phipson B., Wu D., Hu Y., Law C.W., Shi W., Smyth G.K. (2015). limma powers differential expression analyses for RNA-sequencing and microarray studies. Nucleic Acids Res..

[25] Bourdakou M.M., Melliou E., Magiatis P., Spyrou G.M. (2024). Computational investigation of the functional landscape of the protective role that extra virgin olive oil consumption may have on chronic lymphocytic leukemia. J. Transl. Med..

[26] Kim S., Chen J., Cheng T., Gindulyte A., He J., He S., Li Q., Shoemaker B.A., Thiessen P.A., Yu B. (2021). PubChem in 2021: New data content and improved web interfaces. Nucleic Acids Res..

[27] Daina A., Michielin O., Zoete V. (2017). SwissADME: A free web tool to evaluate pharmacokinetics, drug-likeness and medicinal chemistry friendliness of small molecules. Sci. Rep..

[28] Osama A., Zhang J., Yao J., Yao X., Fang J. (2020). Nrf2: A dark horse in Alzheimer’s disease treatment. Ageing Res. Rev..

[29] Robledinos-Anton N., Fernandez-Gines R., Manda G., Cuadrado A. (2019). Activators and Inhibitors of NRF2: A Review of Their Potential for Clinical Development. Oxid. Med. Cell. Longev..

[30] Knox C., Wilson M., Klinger C.M., Franklin M., Oler E., Wilson A., Pon A., Cox J., Chin N.E.L., Strawbridge S.A. (2024). DrugBank 6.0: The DrugBank Knowledgebase for 2024. Nucleic Acids Res..

[31] Corsello S.M., Bittker J.A., Liu Z., Gould J., McCarren P., Hirschman J.E., Johnston S.E., Vrcic A., Wong B., Khan M. (2017). The Drug Repurposing Hub: A next-generation drug library and information resource. Nat. Med..

[32] O’Boyle N.M., Banck M., James C.A., Morley C., Vandermeersch T., Hutchison G.R. (2011). Open Babel: An open chemical toolbox. J. Cheminform..

[33] Cao D.S., Xiao N., Xu Q.S., Chen A.F. (2015). Rcpi: R/Bioconductor package to generate various descriptors of proteins, compounds and their interactions. Bioinformatics.

[34] Sieberts S.K., Perumal T.M., Carrasquillo M.M., Allen M., Reddy J.S., Hoffman G.E., Dang K.K., Calley J., Ebert P.J., Eddy J. (2020). Large eQTL meta-analysis reveals differing patterns between cerebral cortical and cerebellar brain regions. Sci. Data.

[35] Tesi N., van der Lee S., Hulsman M., van Schoor N.M., Huisman M., Pijnenburg Y., van der Flier W.M., Reinders M., Holstege H. (2024). Cognitively healthy centenarians are genetically protected against Alzheimer’s disease. Alzheimer’s Dement..

[36] Amin F.U., Shah S.A., Kim M.O. (2017). Vanillic acid attenuates Abeta(1-42)-induced oxidative stress and cognitive impairment in mice. Sci. Rep..

[37] Pojero F., Aiello A., Gervasi F., Caruso C., Ligotti M.E., Calabro A., Procopio A., Candore G., Accardi G., Allegra M. (2022). Effects of Oleuropein and Hydroxytyrosol on Inflammatory Mediators: Consequences on Inflammaging. Int. J. Mol. Sci..

[38] Sun K.H., Lee H.G., Smith M.A., Shah K. (2009). Direct and indirect roles of cyclin-dependent kinase 5 as an upstream regulator in the c-Jun NH2-terminal kinase cascade: Relevance to neurotoxic insults in Alzheimer’s disease. Mol. Biol. Cell..

[39] Yadav E., Yadav P., Khan M.M.U., Singh H., Verma A. (2022). Resveratrol: A potential therapeutic natural polyphenol for neurodegenerative diseases associated with mitochondrial dysfunction. Front. Pharmacol..

[40] Banji O.J.F., Banji D., Makeen H.A., Alqahtani S.S., Alshahrani S. (2022). Neuroinflammation: The Role of Anthocyanins as Neuroprotectants. Curr. Neuropharmacol..

[41] Kumju Youn C.-T.H. (2022). Mira Jun, Multifaceted neuroprotective effects of (-)-epigallocatechin-3-gallate (EGCG) in Alzheimer’s disease: An overview of pre-clinical studies focused on β-amyloid peptide. Food Sci. Hum. Wellness.

[42] Sano M., Ernesto C., Thomas R.G., Klauber M.R., Schafer K., Grundman M., Woodbury P., Growdon J., Cotman C.W., Pfeiffer E. (1997). A controlled trial of selegiline, alpha-tocopherol, or both as treatment for Alzheimer’s disease. The Alzheimer’s Disease Cooperative Study. N. Engl. J. Med..

[43] Gugliandolo A., Chiricosta L., Silvestro S., Bramanti P., Mazzon E. (2019). alpha-Tocopherol Modulates Non-Amyloidogenic Pathway and Autophagy in an In Vitro Model of Alzheimer’s Disease: A Transcriptional Study. Brain Sci..

[44] Garcia-Garcia V.A., Alameda J.P., Page A., Casanova M.L. (2021). Role of NF-kappaB in Ageing and Age-Related Diseases: Lessons from Genetically Modified Mouse Models. Cells.

[45] Serreli G., Deiana M. (2020). Extra Virgin Olive Oil Polyphenols: Modulation of Cellular Pathways Related to Oxidant Species and Inflammation in Aging. Cells.

[46] Zheng Y., Zhang X., Zhang R., Wang Z., Gan J., Gao Q., Yang L., Xu P., Jiang X. (2023). Inflammatory signaling pathways in the treatment of Alzheimer’s disease with inhibitors, natural products and metabolites (Review). Int. J. Mol. Med..

[47] Reis e Sousa C., Yamasaki S., Brown G.D. (2024). Myeloid C-type lectin receptors in innate immune recognition. Immunity.

[48] Almeida-da-Silva C.L.C., Savio L.E.B., Coutinho-Silva R., Ojcius D.M. (2023). The role of NOD-like receptors in innate immunity. Front. Immunol..

[49] Kawai T., Akira S. (2010). The role of pattern-recognition receptors in innate immunity: Update on Toll-like receptors. Nat. Immunol..

[50] Geijtenbeek T.B., Gringhuis S.I. (2009). Signalling through C-type lectin receptors: Shaping immune responses. Nat. Rev. Immunol..

[51] O’Shea J.J., Murray P.J. (2008). Cytokine signaling modules in inflammatory responses. Immunity.

[52] Medzhitov R. (2001). Toll-like receptors and innate immunity. Nat. Rev. Immunol..

[53] Chen Z., Balachandran Y.L., Chong W.P., Chan K.W.Y. (2024). Roles of Cytokines in Alzheimer’s Disease. Int. J. Mol. Sci..

[54] Ransohoff R.M. (2009). Chemokines and chemokine receptors: Standing at the crossroads of immunobiology and neurobiology. Immunity.

[55] Wang H., Zong Y., Zhu L., Wang W., Han Y. (2023). Chemokines in patients with Alzheimer’s disease: A meta-analysis. Front. Aging Neurosci..

[56] Linzer N., Trumbull A., Nar R., Gibbons M.D., Yu D.T., Strouboulis J., Bungert J. (2021). Regulation of RNA Polymerase II Transcription Initiation and Elongation by Transcription Factor TFII-I. Front. Mol. Biosci..

[57] Balez R., Steiner N., Engel M., Munoz S.S., Lum J.S., Wu Y., Wang D., Vallotton P., Sachdev P., O’Connor M. (2016). Neuroprotective effects of apigenin against inflammation, neuronal excitability and apoptosis in an induced pluripotent stem cell model of Alzheimer’s disease. Sci. Rep..

[58] Rita L., Neumann N.R., Laponogov I., Gonzalez G., Veselkov D., Pratico D., Aalizadeh R., Thomaidis N.S., Thompson D.C., Vasiliou V. (2023). Alzheimer’s disease: Using gene/protein network machine learning for molecule discovery in olive oil. Hum. Genom..

[59] Ansari M.A., Abdul H.M., Joshi G., Opii W.O., Butterfield D.A. (2009). Protective effect of quercetin in primary neurons against Abeta(1-42): Relevance to Alzheimer’s disease. J. Nutr. Biochem..

[60] Zaplatic E., Bule M., Shah S.Z.A., Uddin M.S., Niaz K. (2019). Molecular mechanisms underlying protective role of quercetin in attenuating Alzheimer’s disease. Life Sci..

[61] Ahmad S., Jo M.H., Ikram M., Khan A., Kim M.O. (2021). Deciphering the Potential Neuroprotective Effects of Luteolin against Abeta(1)-(42)-Induced Alzheimer’s Disease. Int. J. Mol. Sci..

[62] Servili M., Sordini B., Esposto S., Urbani S., Veneziani G., Di Maio I., Selvaggini R., Taticchi A. (2013). Biological Activities of Phenolic Compounds of Extra Virgin Olive Oil. Antioxidants.

[63] Manach C., Scalbert A., Morand C., Remesy C., Jimenez L. (2004). Polyphenols: Food sources and bioavailability. Am. J. Clin. Nutr..

[64] Jimenez-Lopez C., Carpena M., Lourenco-Lopes C., Gallardo-Gomez M., Lorenzo J.M., Barba F.J., Prieto M.A., Simal-Gandara J. (2020). Bioactive Compounds and Quality of Extra Virgin Olive Oil. Foods.

[65] Sayed-Ahmad B.U.M., Hijazi A., Saad Z., Cerny M., Evon P., Talou T., Merah O. (2022). Amaranth Oilseed Composition and Cosmetic Applications. Separations.

[66] Ryan E., Galvin K., O’Connor T.P., Maguire A.R., O’Brien N.M. (2007). Phytosterol, squalene, tocopherol content and fatty acid profile of selected seeds, grains, and legumes. Plant Foods Hum. Nutr..

[67] Kim S.K., Karadeniz F. (2012). Biological importance and applications of squalene and squalane. Adv. Food Nutr. Res..

[68] Zhao L., Wang J.L., Liu R., Li X.X., Li J.F., Zhang L. (2013). Neuroprotective, anti-amyloidogenic and neurotrophic effects of apigenin in an Alzheimer’s disease mouse model. Molecules.

[69] Ali Mohammadkhanizadeh M.S. (2025). Soroush Taherkhani, Davood Nourabadi, Seyed Mahdi Mohamadi-Zarch, Farnaz Nikbakht, Yaser Azizi, Protective effects of apigenin in neurodegeneration: An update on the potential mechanisms. Brain Disord..

[70] He Z., Li X., Wang Z., Cao Y., Han S., Li N., Cai J., Cheng S., Liu Q. (2023). Protective effects of luteolin against amyloid beta-induced oxidative stress and mitochondrial impairments through peroxisome proliferator-activated receptor gamma-dependent mechanism in Alzheimer’s disease. Redox Biol..

[71] Kabir E.R., Chowdhury N.M., Yasmin H., Kabir M.T., Akter R., Perveen A., Ashraf G.M., Akter S., Rahman M.H., Sweilam S.H. (2023). Unveiling the Potential of Polyphenols as Anti-Amyloid Molecules in Alzheimer’s Disease. Curr. Neuropharmacol..

[72] Chang W., Huang D., Lo Y.M., Tee Q., Kuo P., Wu J.S., Huang W., Shen S. (2019). Protective Effect of Caffeic Acid against Alzheimer’s Disease Pathogenesis via Modulating Cerebral Insulin Signaling, beta-Amyloid Accumulation, and Synaptic Plasticity in Hyperinsulinemic Rats. J. Agric. Food Chem..

[73] Azargoonjahromi A., Abutalebian F. (2024). Unraveling the therapeutic efficacy of resveratrol in Alzheimer’s disease: An umbrella review of systematic evidence. Nutr. Metab..

[74] Ramassamy C. (2006). Emerging role of polyphenolic compounds in the treatment of neurodegenerative diseases: A review of their intracellular targets. Eur. J. Pharmacol..

[75] Spencer J.P. (2007). The interactions of flavonoids within neuronal signalling pathways. Genes. Nutr..

[76] Chen Z., Zhong C. (2014). Oxidative stress in Alzheimer’s disease. Neurosci. Bull..

[77] Ganguly G., Chakrabarti S., Chatterjee U., Saso L. (2017). Proteinopathy, oxidative stress and mitochondrial dysfunction: Cross talk in Alzheimer’s disease and Parkinson’s disease. Drug Des. Devel. Ther..

[78] Zhou J., Chao G., Li Y., Wu M., Zhong S., Feng Z. (2016). Activation of NRF2/ARE by isosilybin alleviates Abeta25-35-induced oxidative stress injury in HT-22 cells. Neurosci. Lett..

[79] Ramsey C.P., Glass C.A., Montgomery M.B., Lindl K.A., Ritson G.P., Chia L.A., Hamilton R.L., Chu C.T., Jordan-Sciutto K.L. (2007). Expression of Nrf2 in neurodegenerative diseases. J. Neuropathol. Exp. Neurol..

[80] Manach C., Williamson G., Morand C., Scalbert A., Remesy C. (2005). Bioavailability and bioefficacy of polyphenols in humans. I. Review of 97 bioavailability studies. Am. J. Clin. Nutr..

[81] Scalbert A., Manach C., Morand C., Remesy C., Jimenez L. (2005). Dietary polyphenols and the prevention of diseases. Crit. Rev. Food Sci. Nutr..

[82] Kamatham P.T., Shukla R., Khatri D.K., Vora L.K. (2024). Pathogenesis, diagnostics, and therapeutics for Alzheimer’s disease: Breaking the memory barrier. Ageing Res. Rev..

